# Microbial Profiling of a Suppressiveness-Induced Agricultural Soil Amended with Composted Almond Shells

**DOI:** 10.3389/fmicb.2016.00004

**Published:** 2016-01-22

**Authors:** Carmen Vida, Nuria Bonilla, Antonio de Vicente, Francisco M. Cazorla

**Affiliations:** Departamento de Microbiología, Facultad de Ciencias, Instituto de Hortofruticultura Subtropical y Mediterránea “La Mayora”, Universidad de Málaga, Consejo Superior de Investigaciones CientíficasMálaga, Spain

**Keywords:** soil, amendment, almond shells, microbial profiling, suppressiveness

## Abstract

This study focused on the microbial profile present in an agricultural soil that becomes suppressive after the application of composted almond shells (AS) as organic amendments. For this purpose, we analyzed the functions and composition of the complex communities present in an experimental orchard of 40-year-old avocado trees, many of them historically amended with composted almond shells. The role of microbes in the suppression of *Rosellinia necatrix*, the causative agent of avocado white root rot, was determined after heat-treatment and complementation experiments with different types of soil. Bacterial and fungal profiles obtained from natural soil samples based on the 16S rRNA gene and ITS sequencing revealed slight differences among the amended (AS) and unamended (CT) soils. When the soil was under the influence of composted almond shells as organic amendments, an increase in *Proteobacteria* and *Ascomycota* groups was observed, as well as a reduction in *Acidobacteria* and *Mortierellales*. Complementary to these findings, functional analysis by GeoChip 4.6 confirmed these subtle differences, mainly present in the relative abundance of genes involved in the carbon cycle. Interestingly, a group of specific probes included in the “soil benefit” category was present only in AS-amended soils, corresponding to specific microorganisms previously described as potential biocontrol agents, such as *Pseudomonas* spp., *Burkholderia* spp., or *Actinobacteria*. Considering the results of both analyses, we determined that AS-amendments to the soil led to an increase in some orders of *Gammaproteobacteria, Betaproteobacteria*, and *Dothideomycetes*, as well as a reduction in the abundance of *Xylariales* fungi (where *R. necatrix* is allocated). The combination of microbial action and substrate properties of suppressiveness are discussed.

## Introduction

The enhancement of soil suppressiveness using organic amendments has been widely described, especially for soil-borne diseases (Lazarovits et al., 2001; Bailey and Lazarovits, 2003; van Elsas and Postma, 2007; Bonilla et al., 2012b; Pane et al., 2013). However, this effect can be extremely variable depending on the pathosystem and the environmental conditions, and there are even some examples of the amendment application increasing disease incidence (Termorshuizen et al., 2006; Janvier et al., 2007). The soils that become suppressive soils provide an environment in which plant disease development is reduced, even in the presence of a virulent pathogen and a susceptible host (Hadar and Papadopoulou, 2012). This phenomenon could be induced as a direct result of the activity of microorganism consortia that are naturally established on soil after application of the amendment (Weller et al., 2002).

As such, understanding the diversity, composition, structure, function and interactions of microbial communities is crucial to gain insight into the basis for suppressiveness mediated by this organic amendment (Janvier et al., 2007). Approaches for studying microbial communities in the soil are complex. Thus, employing genomic approaches to understand which changes occur in soil could be a good alternative strategy to decipher the profiling of soil microbiota (Garbeva et al., 2004).

The use of genomic techniques rely on PCR amplification of the conserved and variable regions of the microbial genome, commonly 16S ribosomal RNA (rRNA) for bacteria and 18S rRNA or internal transcribed sequences (ITS) for fungi, allowing for direct sequencing of these PCR amplicons using different high-throughput next-generation sequencing methods. Each group of PCR amplicons that shares a similar or identical variable region is considered an “operational taxonomic unit” (OTU) and is assumed to be equivalent to a microbial species or genus. The analysis of OTUs provide information about the phylogenetic diversity of the soil microbial community (van Elsas et al., 2007, 2008; Hirsch et al., 2013; Koyama et al., 2014).

Moreover, complementary techniques have arisen, such as microarrays, which have considerable potential in environmental microbial ecology, providing novel insights into how environmental factors affect microbial communities in various habitats (Hazen et al., 2010; He et al., 2012; Bai et al., 2013; Zhang et al., 2013; Tu et al., 2014). The GeoChip microarray is a comprehensive functional gene array (FGA) targeting hundreds to thousands of different gene families that play important roles in various biogeochemical processes, enabling researchers to comprehensively analyse the functional diversity, composition, and structure of microbial communities in various environments. It is a powerful FGA-based technology that can be used to survey the functional diversity, composition, structure, metabolic potential/activity, and dynamics of microbial communities, and then link them with ecosystem processes and functions (Xie et al., 2011; Xue et al., 2013; Cong et al., 2015).

Our research interest is focused on the avocado (*Persea americana* Mill.), for which southern Spain is one of the most relevant zones in the Mediterranean area for this crop. In this part of the world, one of the most limiting soilborne diseases affecting avocado trees is white root rot, caused by the fungus *R. necatrix* Prill. White root rot is considered to be an emergent threat to many woody crop plants worldwide (Pliego et al., 2009, 2012).

The role of soil microorganisms in the plant protection have been broadly reported. Thus, different microbes can contribute to the biocontrol of avocado white root rot using different weaponry such as antagonism (*Pseudomonas chlororaphis* PCL1606 or *Bacillus subtilis* PCL1608; Cazorla et al., 2006, 2007), competition for niches and nutrients (Calderón et al., 2014), or induction of systemic resistance or predation (*Trichoderma* spp.; Ruano-Rosa and López-Herrera, 2009). These microorganisms can act as single or combined with other biocontrol agents against *R. necatrix* (Ruano-Rosa et al., 2014). Other studies have reported the positive effect of the application of arbuscular mycorrhizal fungi to soil and the biocontrol activity on avocado (Hass and Menge, 1990; González-Cortés et al., 2012).

During the past decades, several approaches have been implemented to achieve an integrated management of *R. necatrix*, including physical, chemical and biological control approaches (López-Herrera et al., 1998; López-Herrera and Zea-Bonilla, 2007; González-Sánchez et al., 2013). All of these approaches seem to be effective at the experimental level, and some of them have been proven to be effective under certain conditions. However, at the same time, traditional strategies of land management have improved, and some of these strategies could be considered useful approaches to fight against diseases in avocado management, thus increasing the weaponry available against white root rot (Bonilla et al., 2012b).

One of these approaches is the use of organic amendments or mulches, which have produced beneficial effects for plants, including increasing health and yields in avocado crops (Moore-Gordon et al., 1997; Wolstenholme et al., 1997; Hermoso et al., 2011). It has been previously shown that the application of such organic matter to avocado agricultural soil can affect soil physicochemical properties and microbial communities (Bonilla et al., 2012a; López et al., 2014). Additionally, organic amendments could play a critical role in global biochemical cycles (Bonanomi et al., 2014) and could cause different effects, such as the improvement of soil fertility and the enhancement of natural suppressiveness of the soil against several phytopathogens (Cretoiu et al., 2013). Several organic amendments have shown an obvious suppressive effect against another important avocado soil-borne phytopathogen, *Phytophthora cinnamomi* (Bender et al., 1992; Downer et al., 2001).

In a previous study, it was shown that different organic matter applied as a mulch to the avocado crop exhibited suppressive effects against white root rot (Bonilla et al., 2015). Composted almond shells were one type of organic matter tested. The application of composted almond shells as a mulch led to an enhancement of the bacterial composition and activities of the soil communities in relation to the observed suppressiveness (Bonilla et al., 2015).

The objective of the present study was to gain insight into the microbial profiling present in the amended soils showing suppressive ability against the avocado soil-borne phytopathogen *R. necatrix*. The use of different microbial approaches should uncover the microbial communities potentially involved in the suppressive phenotype.

## Materials and methods

### Field of study

Soil samples were obtained from an avocado crop field (cv. *Hass* avocado trees grafted onto cv. *Topa-Topa* seedling rootstocks) located at the Experimental Station “La Mayora” (IHSM-UMA-CSIC, Málaga, Spain) on the coast of the Malaga Province (SE Spain). This experimental field of 2.5 km^2^ (36°75′N, 4°04′O) contains 195 40-year-old avocado trees planted at 8 × 8 m. Selected avocado trees were grouped in pairs to facilitate their management. Sixteen pairs of trees were under ecological management (massive application of composted almond shells in 2002, 2007, and 2012), and another 16 pairs of trees were under conventional management (addition of mineral nutrients twice per year, as well as the application of herbicides and pesticides when necessary, López et al., 2014) and without any organic amendment.

### Soil sampling

Natural field soil samples allocated underneath of avocado trees unamended (CT) or amended with composted almond shells (AS) were taken to perform the different experiments. Soil samples were collected in April 2013, November 2013 and April 2014. Composite soil samples were taken from four different groups of paired trees with (AS) or without (CT) organic amendment and were randomly selected from throughout the avocado orchard. To obtain a composite soil sample, two sampling distal points at 1.5 m around the trunk base for each tree of a pair of trees under the same treatment were selected; the upper layer of compost was carefully removed, and 5–10 kg of soil samples (15 cm depth) were collected per pair of trees and merged. Samples were placed in cold storage and transported to the laboratory. Samples of each type of soil were sieved through a 20 mm mesh and immediately used for physicochemical and suppressiveness experiments. To perform DNA extractions, three soil samples (1 g each) from composite soil samples per each pair of trees were sieved again (2 mm diameter) and processed independently. The remaining unused soil samples were stored at −80°C.

### Physicochemical analysis of soil samples

Physicochemical analysis of both types of soil samples were performed at Laboratorio Caisur S.L. (Granada, Spain) using standardized methodologies. Four samples from each composite field soil sample (AS and CT) were analyzed independently.

### Soil processing

**Figure 1:**
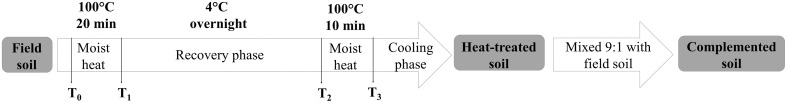
**Processing scheme of the soil heat-treatment and complementation used in this study for the agricultural field soil samples**. The same procedure was followed for both unamended soil and soil amended with composted almond shells. T_0–3_ indicates sampling points to perform bacterial and fungal plate counts.

To test the potential role of soil microorganisms in suppressiveness, we prepared three types of processed soils using different treatments: Field soils (raw soils), heat-treated soils, and complemented soils (Table 1). We applied a moist heat treatment to the field soil samples as previously described (Weller et al., 2002), with slight modifications. Briefly, the heat treatment consisted of heating the soil in high moisture conditions at 100°C for 20 min in an autoclave. The soil was allowed to recover at 4°C overnight. Then, we performed a second treatment step, heating the soil at 100°C for 10 min in high moisture conditions. After allowing it to cool, the soil was ready to be used (Figure 1). Complemented soils were prepared with the purpose of observing the partial recovery of the microbial characteristics of the natural soil (Weller et al., 2002). The complemented soil consisted of heat-treated soil mixed with natural raw field soil in a 9:1 (w/w) ratio (Table 1).

**Table 1:** **Types of processed agricultural soils used in this study**.

**Soil source**	**Treatment code**	**Details of processed soils**
Amended with composted almond shells	AS	Natural field soil amended with composted almond shells mulching
	ASt	AS heat-treated soil
	ASc	ASt complemented with AS in 9:1 (w/w) ratio
	ASt+CT	ASt complemented with CT in 9:1 (w/w) ratio
Unamended and under conventional management	CT	Natural field soil unamended and under conventional management
	CTt	CT heat-treated soil
	CTc	CTt complemented with CT in 9:1 (w/w) ratio
	CTt+AS	CTt complemented with AS in 9:1 (w/w) ratio

A scheme of the processing is described in Figure 1.

To evaluate changes in the culturable microbiota fraction during different times of the soil sample processing, counts of cultivable colony forming units (CFUs) of bacteria and fungi per gram of soil were performed. For this, 2 g samples of soil obtained at the different key times during the process were suspended in 20 ml of sterile saline solution (0.85% NaCl) with 0.5 g of sterile gravel and mixed at 150 rpm for 30 min on an orbital shaker at room temperature. Ten-fold serial dilutions of the obtained suspensions were plated on Luria Bertani (LB) agar with 100 mg of cycloheximide per liter, to analyse the heterotrophic bacteria group, and on potato dextrose agar (PDA) with 50 mg of chlortetracycline and 1 ml of tergitol NP-10 (Sigma) per liter (Bonilla et al., 2012a).

### Suppressiveness assays

Suppressiveness assays against white root rot caused by the virulent strain *R. necatrix* CH53 (López-Herrera and Zea-Bonilla, 2007) were conducted using two different susceptible pathosystems, avocado (Cazorla et al., 2006) and wheat (*Triticum aestivum*). The *R. necatrix* inoculum was produced on wheat seeds (Freeman et al., 1986). The seeds were soaked for 12 h in 250-ml Erlenmeyer flasks filled with distilled water. The flasks were autoclaved after excess water had been drained off. After sterilization, fungal disks of a 1-week-old culture of *R. necatrix* grown on PDA were placed aseptically in each flask. Flasks were incubated at 25°C for 2–3 weeks and were shaken every 2–3 days to avoid clustering of the seeds.

#### Avocado/*R. necatrix* test system

Six-month-old commercial avocado plants were obtained from Brokaw nurseries (Brokaw España, S.L., Vélez-Málaga, Spain). The roots from the avocado plants were disinfected by immersion in 0.1% NaOCl for 20 min and then washed twice (20 min) with sterile distilled water. Then, avocado plants were placed into square plastic pots (10.5 × 10.5 × 10.5 cm) containing 0.64 L of the sieved CT and AS types of soils. Fungal infection with *R. necatrix* was performed using wheat grains (4 holes of 2 cm depth were made per pot, 3 infected wheat grains were placed per hole) as previously described (Freeman et al., 1986). Non-infected plants were used as controls. Three sets of 15 avocado plants were tested per type of soil. The plants were grown in a chamber at 25°C with 70% relative humidity and 16 h of daylight and were watered twice per week. Aerial symptoms of avocado white root rot were recorded on a scale of 0–3, and a disease index (DI) was calculated after 5 weeks using the previously described formula (Cazorla et al., 2006).

#### Wheat/*R. necatrix* test system

Wheat seeds were disinfected by immersion in 0.05% NaOCl for 10 min, washed and then placed in darkness between pieces of moist filter paper in a growth chamber for 2–3 days at 25°C to induce germination. Then, germinated seedlings were disinfected again by immersion in 0.1% NaOCl for 20 min and washed (20 min) with sterile distilled water. Seedlings were placed into plastic seedling trays (5 cm diameter × 5.5 cm) containing 0.08 L of different types of soils and either infected with *R. necatrix* using wheat grains (three grains per slot) or not infected to be used as controls. Three sets of 50 wheat seedlings were tested per type of soil. The seedlings were grown in a chamber at 25°C with 70% relative humidity and 16 h of daylight and were watered twice per week. Aerial symptoms were evaluated, and the disease index percentage was calculated as previously described for the avocado/*R. necatrix* system (Cazorla et al., 2006). Disease index percentage was recorded after evaluation of symptoms, with values ranging between 0 (healthy plant), 1 (yellowing stem base), 2 (drying stem base), and 3 (dead plant). The number of diseased seedlings was determined 7 weeks after beginning the assay, and the disease index was calculated as previously described (Cazorla et al., 2006).

### Soil DNA extraction

Soil DNA extraction was performed using 1.0 g of soil samples and a PowerSoil® DNA Isolation Kit (MOBIO Laboratories, Inc, Carlsbad, CA, USA). DNA was extracted from three independent soil samples per pair of trees for amended and unamended soil (AS and CT) and checked for quality. To test the DNA quality we performed a DNA digestion using the restriction enzyme *Eco*RI (New England BioLabs®, Inc., Ipswich, MA, UK) and PCR amplification of the variable region of the bacterial 16S rDNA with the universal bacterial primers 341F and 907R as described by Muyzer et al. (2004). Digestion and PCR products were analyzed for size by agarose gel electrophoresis and ethidium bromide staining. Suitable samples were mixed and DNA quantity and quality (A_260_/A_230_ > 1.8 and A_260_/A_280_ > 1.7) were evaluated using a NanoDrop ND-1000 spectrophotometer (NanoDrop Technologies Inc., Wilmington, DE, USA).

Three independent DNA extractions were performed per each pair of trees, and then merged to create a composite DNA sample. Three of these composite DNA extractions were independently analyzed for each type of field soil (AS and CT). DNA was stored at −20°C for further analyses.

### Analysis of 16S rRNA and its gene sequence

Two composite DNA samples from each soil type were sent for sequencing by STAB VIDA (NGS Laboratories, Caparica, Portugal) and sent to ChunLab (Seoul, Korea) to obtain the microbial DNA sequences of the 16S rRNA gene and ITS hypervariable regions. Sequences were analyzed using QIIME software (Caporaso et al., 2010) and CLcommunity™ software (ChunLab). Sequences of a length less than 200 nt were excluded from the analysis. The data were filtered for noisy sequences, checked for the presence of chimeras, and binned into OTUs (Peiffer et al., 2013) at the 97% sequence similarity level. A representative sequence of each OTU was taxonomically classified. The relative abundance of microbial clades at different taxonomic levels was calculated as the average value from two independent analyses and was used to perform the comparative distribution analysis.

### Geochip analysis

Three of the composite samples of purified test DNA (800 ng per sample) from the two different types of soils studied (AS and CT) were sent to Glomics Inc (Norman, Oklahoma) for the sequencing analysis (Tu et al., 2014). Briefly, after the hybridization steps, the arrays were washing, dried and then scanned. The images obtained were analyzed by NimbleScan software (Roche NimbleGen Inc., Madison, WI) using the gridding file containing GeoChip 4.6 probes and NimbleGen control probes to determine the intensity of each spot and to identify low quality spots, which were removed prior to statistical analysis (probe spots with coefficient of variance > 0.8 were removed). Extracted data were then loaded into the GeoChip data analysis pipeline at the Institute for Environmental Genomics (Microarray Data Manager, http://ieg.ou.edu/microarray/; Liang et al., 2010; Deng and Zhou, 2013). First, the average signal intensity of the common oligo reference standard (CORS) was calculated for each array, and the maximum average value was applied to normalize the signal intensity of samples in each array. Second, the sum of the signal intensity of the samples was calculated for each array, and the maximum sum value was applied to normalize the signal intensity of all of the spots on an array, which produced a normalized value for each spot in each array. Spots were scored as positive based on a floating signal-to-noise ratio [SNR = (signal mean–background mean)/background standard deviation] so that hyperthermophile control probes accounted for 5% of positive probes. Spots that were detected in less than two samples were also removed. Before statistical analysis, logarithmic transformation was carried out for the remaining spots, and the signals of all spots were transformed into relative abundances (the sum of the number of hybridized probes for each gene category or gene function between the number of total detected probes).

Data processing was used for further analyses. Genes that overlapped between treatments (AS and CT) were calculated by dividing the number of overlapped genes between the treatments by the number of all genes detected in both treatments. Gene function diversity was calculated using the Shannon-Weiner index (H', alpha diversity) and Simpson's index (1/D, beta diversity). We performed a detrended correspondence analysis (DCA) to measure the differences of community functional gene structure between treatments. For comparing the different gene function communities, a hierarchical clustering analysis using Bray-Curtis distances was also performed. To analyse the unique detected probes in the AS samples, we performed a Venn diagram analysis using an on-line tool (http://bioinfogp.cnb.csic.es/tools/venny/). Previously, we prepared two databases by selecting genes (probes) that hybridize exclusively in each type of soil and compared them. This website provided us with a list of 2766 AS unique detected sequences from suppressive soil, which were selected to perform specific comparative analysis.

### Statistical methods

For suppressive analysis, the data were statistically analyzed using an analysis of variance (Sokal and Rohlf, 1986), followed by Fisher's least significant difference test (*P* = 0.05) using SPSS 22 software (SPSS Inc., Chicago). For GeoChip 4.6 analysis, significant differences in relative abundances of the microbial gene diversity between different soils were analyzed by an unpaired Student's *t*-test. A significance level of *P* < 0.1 was adopted for all comparisons. Based on the standard error, the 95% confident interval for each response variable was obtained and the significant differences between the soils were estimated.

## Results

### Characteristics of avocado field soils

The soil samples were taken from the same avocado orchard but from trees under different soil management (AS-amended or unamended). Soil characteristics of the experimental avocado field revealed sandy-loam textures for the amended (AS) and unamended (CT) soils. The pH was not substantially different among these samples and ranged from 7.20 to 7.55 (nearly neutral pH). Some macro- and micro-nutrients, such as potassium, iron and manganese, were also increased in the AS-amended soil (data not shown).

### White root rot suppressiveness assay

Suppressiveness assays against white root rot were performed using the avocado/*R. necatrix* and the wheat/*R. necatrix* experimental plant test systems. AS-amended and unamended avocado agricultural soils, after different experimental heat treatments an complementantions were used (Figure 1; Table 1).

Bacterial and fungal counts of AS-amended and CT soil were very similar, with values of 6.5 and 6.6 log_10_ bacterial cfu/g, respectively, and 5.0 and 5.1 log_10_ fungal cfu/g, respectively. After the heat treatment of the soil, bacterial counts decreased and stabilized, without any further changes after a second heat treatment in any type of soil (Table 2). There were no differences in the results obtained for fungal count (Table 2).

**Table 2:** **Plate counts of total heterotrophic bacteria and fungi during the soil heat-treatment of the unamended and amended with composted almond shells**.

**Plate counts of**	**Soil source sample**	**Sampling points during the heat-treatment process**			
		**T_0_**	**T_1_**	**T_2_**	**T_3_**
Heterotrophic bacteria	AS	6.5 ± 0.48	5.9 ± 0.76	6.0 ± 0.42	5.9 ± 0.59
	CT	6.6 ± 0.30	5.9 ± 0.64	5.9 ± 0.30	5.7 ± 0.64
Heterotrophic fungi	AS	5.0 ± 0.90	4.7 ± 0.67	4.9 ± 0.57	4.7 ± 0.60
	CT	5.1 ± 0.98	4.9 ± 0.55	5.0 ± 0.67	4.8 ± 0.87

T_0–3_ indicates sampling points used along the process. Microbial counts data are presented as log10 cfu/g soil ± standard deviation.

**Figure 2:**
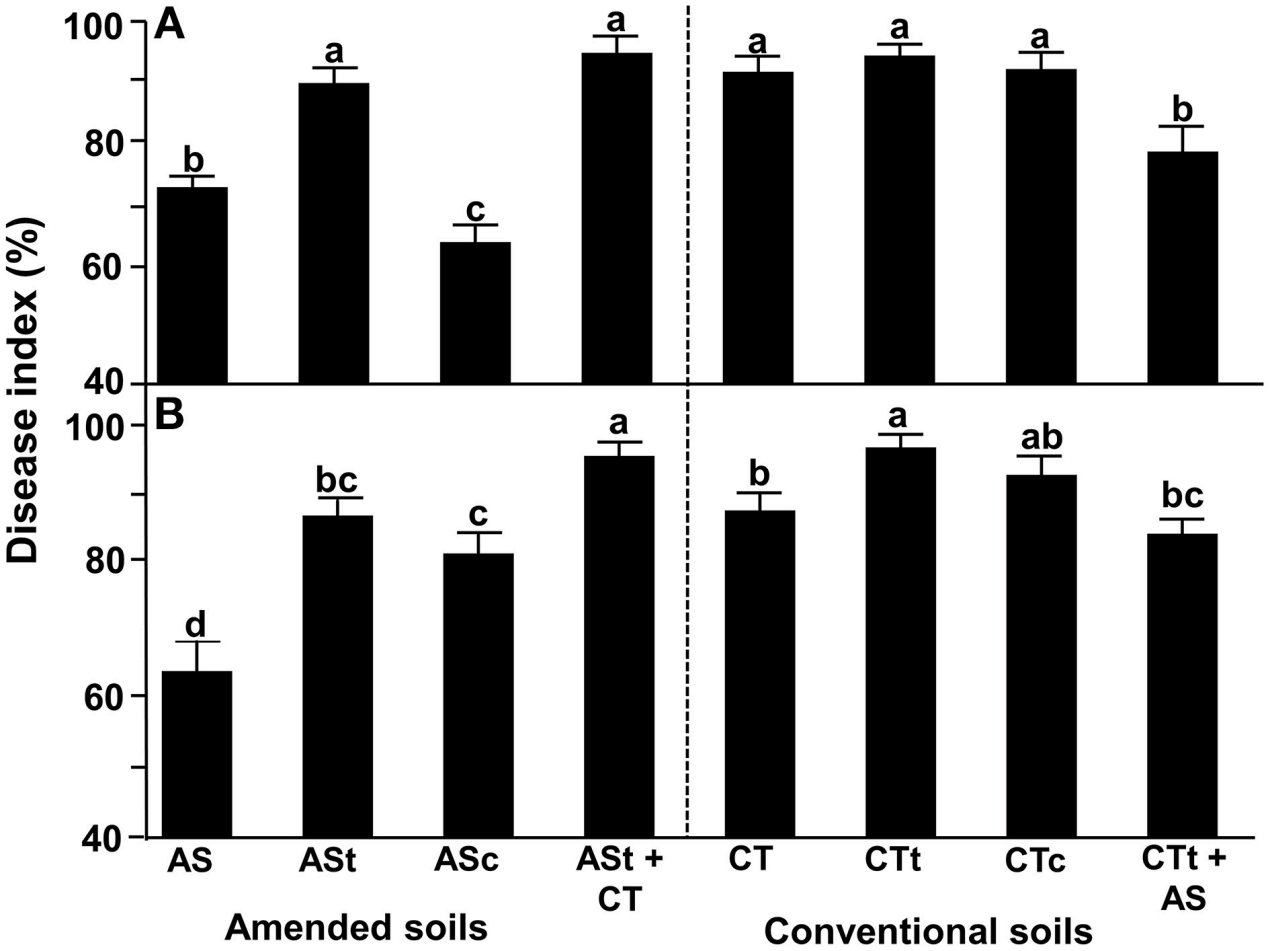
**Suppressiveness assays using the avocado/*R. necatrix* (A) and wheat/*R. necatrix* (B) test systems**. AS, agricultural field soil amended with composted almond shells; ASt, AS heat-treated soil; ASc, ASt complemented with AS in 9:1 (w/w) ratio; ASt+CT, ASt complemented with CT in 9:1 (w/w) ratio; CT, Agricultural field soil under conventional management; CTt, CT heat-treated soil; CTc, CTt complemented with CT in 9:1 (w/w) ratio; CTt+AS, CTt complemented with AS in 9:1 (w/w) ratio. Data were analyzed for significance after arcsine square root transformation with analysis of variance, followed by Fisher's least significant difference test (*P* = 0.05). Values of bars with different letters indications denote a statistically significant difference.

For avocado/*Rosellinia* test system, the disease incidence was evaluated after 5 weeks and at the end of the assay, and the disease index (DI) was calculated (Figure 2A). In these studies, AS field soil samples displayed better suppressive ability than CT field soil samples. Plants growing in the presence of AS-amended soil samples displayed a significantly lower DI than plants cultivated in the presence of CT soil samples at the end of the experiment (Figure 2A). The disease suppressiveness activity was reduced when AS soil samples were heat-treated (ASt) but showed no changes in CTt soil. Moreover, suppressiveness was complemented by soils ASc and CTt+AS, when incorporating AS soil samples. Complemented soil ASt+CT and CTc did not have a disease-suppressive ability, with levels resembling those for the heat-treated unamended soil (Figure 2A).

For the wheat/*R. necatrix* plant test system, disease incidence was tested 7 weeks after inoculation when the disease index (DI) was calculated (Figure 2B). Similar to the results shown by the avocado/*R. necatrix* test system, the AS-amended soil exhibited better suppressive ability than CT soil. The suppressiveness phenotype was significantly lost in heat-treated soils (ASt and CTt) and was partially recovered when we used amended field soil to complement (ASc and CTt+AS). The soils complemented with unamended soil, CTt and ASt+CT, had a disease-suppressive ability similar to that of heat-treated unamended soil (Figure 2B).

### Characterization of the soil microbial community based on 16S rRNA gene and its sequencing

DNA profiling approaches and the sequencing of 16S rRNA and the ITS variable regions of extracted and mixed DNA revealed the relative abundances of microbial clades at different taxonomic levels. However, only the most abundant OTUs were quantified with a level of precision sufficient to perform the comparative distribution analysis due to the high level of OTU richness.

**Figure 3:**
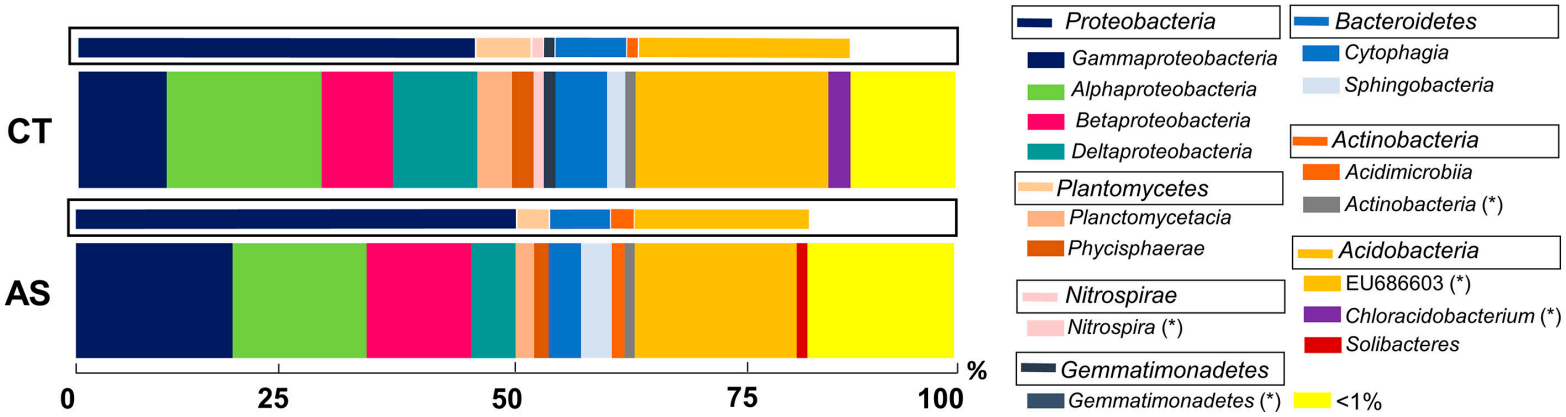
**Analysis of microbial communities present in field soil samples unamended (CT) and amended with composted almond shells (AS)**. Relative abundance (percentage) of different prokaryotic groups detected by 16S rRNA gene sequencing analysis of soil DNA. Analysis of microbial groups are marked at the class level (thick bars) and the phylum level (boxed thin bars). < 1%, sum of all detected groups with a relative abundance less than 1%. ^*^Taxonomic characteristics of these groups are uncertain.

In both samples, *Archaea* were found in a very low relative abundance (< 0.1%). Thus, the bacterial 16S rRNA gene sequences allowed us to identify 33 different representative phyla in AS soil samples and 26 phyla in CT soil samples, from which 5 and 7 phyla comprise more than 1% of the community in AS and CT, respectively (Figure 3).

In AS soil samples, the 5 most abundant phyla (above 89% of relative abundance) were *Proteobacteria* (50.08%), *Acidobacteria* (22.64%), *Bacteroidetes* (8.05%), *Planctomycetes* (4.27%), and *Actinobacteria* (4.09%). In contrast, the analysis of CT soil samples revealed that the most abundant (representing above 95%) phyla were *Proteobacteria* (45.48%), *Acidobacteria* (27.39%), *Bacteroidetes* (8.79%), *Planctomycetes* (60.99%), *Actinobacteria* (3.19%), *Nitrospirae* (1.70%), and *Gemmatimonadetes* (1.63%).

At the class level, the AS soils presented a high abundance of uncultured bacteria from the groups of *Acidobacteria* (EU686603, 18.44%), *Gammaproteobacteria* (17.85%), *Alphaproteobacteria* (15.28%), and *Betaproteobacteria* (11.4%) (Figure 3). In CT soil samples, the class analysis resulted in a similar representation of class abundance, including uncultured bacteria EU686603 (22.99%), *Alphaproteobacteria* (17.7%), and *Gammaproteobacteria* (10.7%).

In both soil samples, the phylum *Proteobacteria* is the most abundant (50.08 and 45.48%). Differences in this group have been shown between the two soil samples. In general, diversity is higher in AS soil samples that exhibit a predominance of the classes *Gammaproteobacteria* (36%) and *Alphaproteobacteria* (30%) and a low percentage of *Deltaproteobacteria*. In CT soil samples, a clear predominance of *Alphaproteobacteria* can be observed (39%). Remarkably, we observed an increase in AS soil samples (almost 2x) of the orders *Steroidobacter* (28%) and *Burkholderiales* (13%) and the decrease of *Rhodospirales* (from 18% in CT to 8% in AS) (Figure S1A).

We observed 76 different classes in AS soil samples and 65 classes in CT soil samples. We detected 24 and 13 specific bacterial classes in AS and CT, respectively, and a slightly higher richness in AS samples (Figure S2A).

The analysis of ITS sequences to reveal the abundance of eukaryotic microbes allowed us to identify a high abundance of fungal microbes. Eukaryotic microbes different from fungi ranged from 7.97% (AS) to 9.52 (CT). Among the fungi detected, the unclassified fungi comprises 8.04% (AS) and 4.28% (CT), and those below 1% represent 2.9% in CT soil samples and 3.4% in AS soil samples.

**Figure 4:**
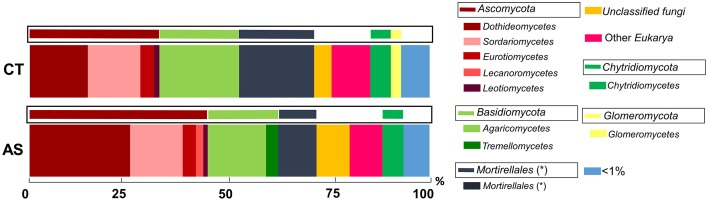
**Analysis of microbial communities present in field soil samples unamended (CT) and amended with composted almond shells (AS)**. Relative abundance (percentage) of different eukaryotic groups detected by ITS region sequence analysis of soil DNA. Analysis of microbial groups are marked at the class level (thick bars) and at phylum level (boxed thin bars). < 1%, sum of all detected groups with a relative abundance less than 1%. ^*^Taxonomic characteristics of these groups are uncertain.

The most abundant fungal groups (approximately 70%) that are in both soil samples are of the phyla *Ascomycota* and *Basidiomycota* and of the group *Mortierellales*. In AS soil samples, an increase in the relative abundance of *Ascomycota* can be observed (Figure 4), (35.37% in CT and 45.79% in AS), as well as a reduction in the group of *Mortierellales* (18.37 in CT and 9.92% in AS).

The analysis of the most abundant group of microorganisms (*Ascomycota*) revealed that in AS soil samples an increase of the class of *Dothideomycetes* (from 40% in CT to 54% in AS) was observed. Additionally, a reduction of the class of *Sordariomycetes* (from 38% in CT to 29% in AS) was observed. Also of note in reference to fungal order in AS soil samples, a huge increase of *Pleosporales* (from 16% in CT to 48% in AS) was observed. Remarkably, one of the fungal order that decreased in AS soil samples was the order *Xylariales* (from 8% in CT to 3% in AS), where the pathogen *R. necatrix* is allocated (Figure S1B).

We observed 39 different classes in AS soil and 50 classes in CT soil. We detected 7 and 18 specific bacterial classes in AS and CT soil, respectively, and observed a slightly higher richness in CT samples (Figure S2B).

### GeoChip analysis in soil samples

The number of total genes detected by GeoChip analysis and overlapping genes between treatments were measured to understand the functional diversity and structure of the microbial communities. The number of total genes detected ranged from 27348 to 28491 and from 29311 to 33526 in AS and CT samples, respectively. An unpaired Student's *t*-test showed that these values were significantly different. The percentage of overlapping genes between samples ranged from 77.18% for AS (77.41, 75.25, and 78.88%) to 73.16% for CT (76.25, 65.70, and 77.52%) (Figure S3). This value fell to 65.43% when we compared overlapping genes between treatments (AS_1–3_ and CT_1–3_). DCA (detrended correspondence analysis) and hierarchical clustering (with Bray-Curtis distance) were performed (Figure S3) using all of the detected genes, showing that functional structure of the microbial community was similar in the replicates but different among the soils (AS and CT).

**Figure 5:**
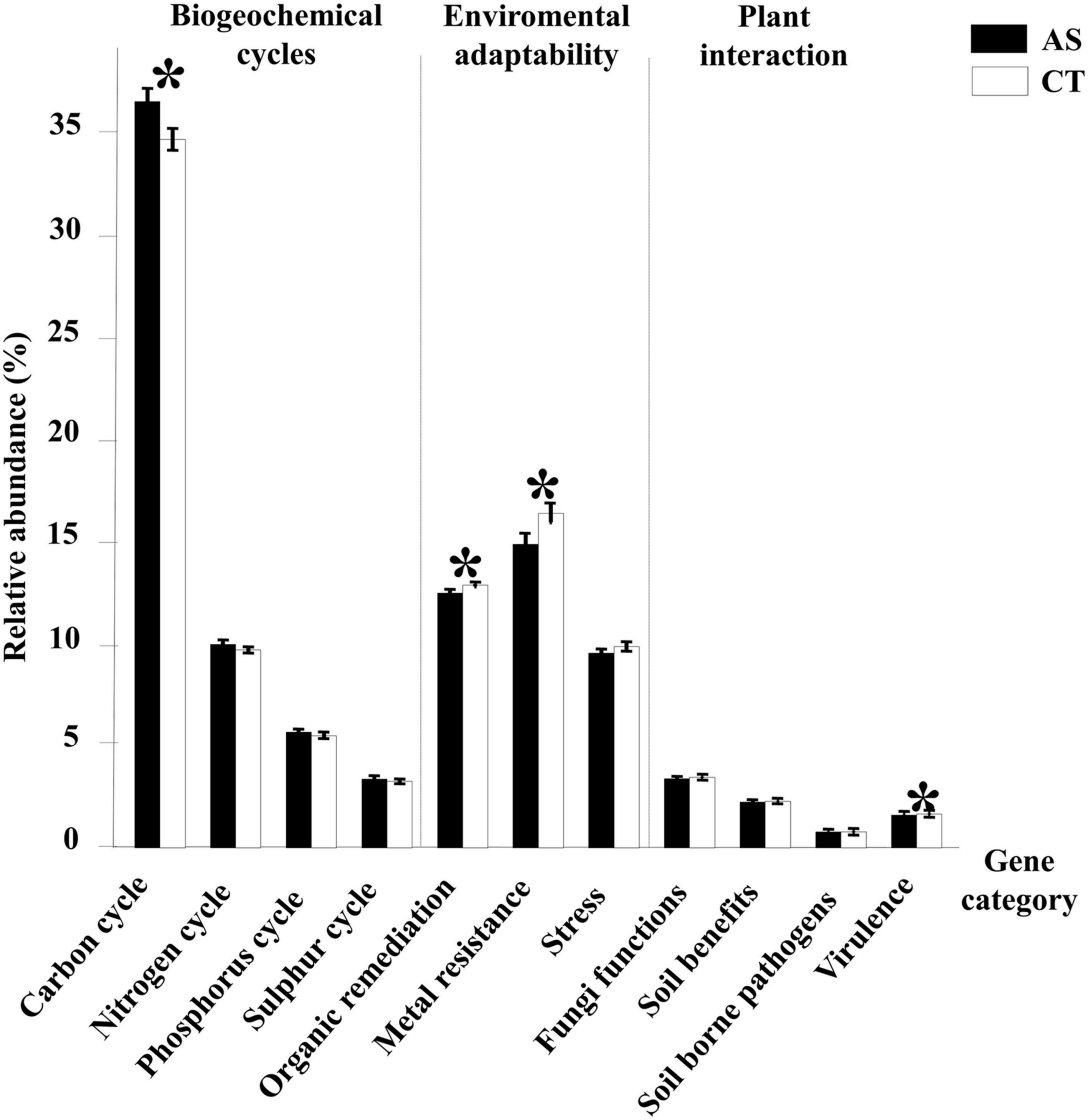
**GeoChip analysis of functional gene categories**. Relative abundance of all detected genes from different gene categories analyzed in this study. ^*^Indicates significant statistical differences (*p* < 0.1) between the two types of soil samples, amended soil (AS) and conventionally managed soil (CT). Standard error bars are shown.

To understand the effects of composted almond shells on the microbial communities and the acquired suppressive capacity, microbial functional genes categorized as participating in biogeochemical cycles and other important soil processes were examined (Figure 5). Gene functions related to the carbon cycle were the gene category most represented in all samples. C cycling probes were significantly more abundant than other categories in AS samples (36.65% in AS and 34.54% in CT), whereas genes related to organic contaminant degradation (12.42% in AS and 12.81% in CT), metal resistance (14.58% in AS and 16.32 in CT) and virulence (1.59% in AS and 1.61% in CT) were significantly more abundant in CT samples. There were no significant differences in N, P, and S cycle genes and other gene categories such as stress, fungi functions, soil benefit and soil borne pathogens (Figure 5).

Key genes for acetogenesis, C degradation, C fixation, methane metabolism, and other genes related to the C cycle were detected in the two types of soils (Figure S4A). The relative abundance of genes related to the C degradation category were the highest and exhibited significant differences between the AS samples and the CT samples. In this category, we found the presence of degradative genes of the most abundant C sources derived from plant and animal sources that could be present in soil ecosystems, such as starch, hemicellulose, cellulose, chitin, and lignin. There were few significant differences between samples in these categories of detected genes (Figure S4A).

Of the nitrogen cycle category, only the ammonification subcategory had a higher significant difference for amended soil (Figure S4B). In this subcategory, there are genes that function in the decomposition of organic matter and cycling of accumulated N.

Related to the sulfur cycle, the analyses performed exhibited a higher significant difference (*P* < 0.1) in only the sulphite reductase genes of AS samples compared to CT samples. These genes encode enzymes that catalyze the reduction of sulphite to sulfide, using iron as cofactor, and provide a source of S to microbiota. The CT samples exhibited a higher significant difference in sulfate reductase, a protein involved in sulfur reduction by anaerobic respiration (Figure S4C).

Statistical analyses showed no significant differences in the relative abundance of genes involved in the phosphorous cycle for these samples.

The analysis of genes in the category of environmental adaptability showed significant differences (*P* < 0.1) in the subcategories, as shown in Figures S4D–F. Genes involved in the organic degradation of aromatics, such as chlorinated and pesticide-related compounds, had a higher significant relative abundance for amended soil than conventional managed soil. Similar results were obtained for genes related to osmotic and oxygen stress, from the stress category, and metal resistance to cobalt and lead, which had slightly higher significant relative abundance for AS samples than CT samples. On the other hand, unamended soils exhibited significantly higher values of relative abundance for genes related with stress induced by glucose limitation and metal resistance to cadmium and other metals.

The category of plant interaction covers a wide range of different functional genes involved in microbial interactions with plants, including genes related to fungal function, soil benefit, soil borne pathogens, and virulence. The analyses performed showed significant differences (*P* < 0.1) in some subcategories, as shown in Figures S4G–J. There were not any significant differences in the genes in the categories of soil benefit or fungi function. Nevertheless, CT samples exhibited a higher significant relative abundance of detected genes from the oomycetes subcategory (soil borne pathogen), which included different genes from this pathogenic group. Genes related to virulence processes such as iron oxidation or secretion had a higher significant relative abundance for amended soils; whereas unamended soils exhibited significantly higher values for genes involved in virulence actions such as iron uptake (aerobactin genes) and pilin formation.

### Unique DNA probes detected in as suppressive soil samples

**Figure 6:**
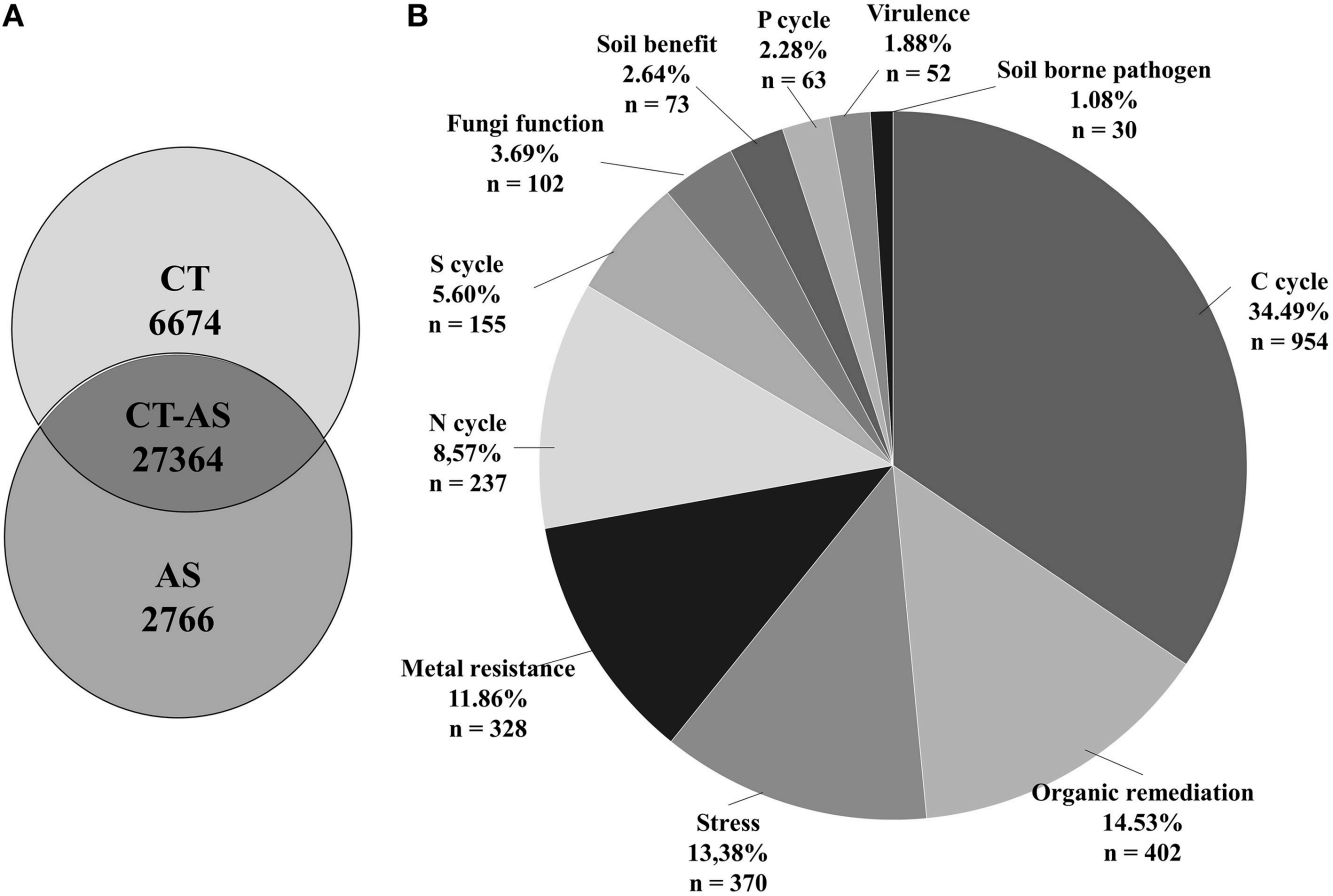
**GeoChip analysis of unique detected genes**. **(A)** Number of core and unique detected genes (different gene ID) of amended soil (AS) and conventionally managed soil (CT). **(B)** Assigned functions of the unique genes detected in the AS sample.

Results of the GeoChip analysis and the Venn diagram representation allowed us to determine microbial specific gene functions detected exclusively in each treatment and the number of commonly detected probes (27364) (Figure 6A). We found 6674 unique detected probes in CT samples and 2766 unique detected probes in AS samples (approximately 10% of the total AS detected genes) from the gene categories analyzed. Approximately 34.49% of the unique hybridizations were related to the Carbon cycle category (Figure 6B), mainly to starch and chitin degradation (Table S1). The Organic remediation gene category exhibited 14.53% unique hybridizations of genes related to the degradation of aromatic compounds. The Stress category had 13.38% unique hybridized probes and the Metal resistance category had 11.86% unique hybridized probes. The Nitrogen cycle category exhibited 8.57% unique hybridized probes, mostly in genes related with denitrification. The remaining gene categories had lower percentages: Sulfur cycle 5.60%, Fungi function 3.69%, Soil benefit 2.64% [approximately 44% of unique detected probes in this category correspond mainly with antimicrobial genes such as *cat* (catalase), *phzF* (phenazine), or *pcbC* (isopenicillin)], Phosphorus cycle 2.28%, Virulence 1.88%, and Soil borne pathogen 1.08% (Figure 6B). This analysis allowed us to relate different gene functions implicated in the metabolism of different soil compounds with bacterial or fungal classes present in the AS soil (Table S1).

## Discussion

The application of organic amendments to agricultural soils is a longstanding practice, and examples of organic-amendment-mediated suppression of soilborne diseases were reported as early as the late nineteenth century (Stone et al., 2004). Growers have observed that different types of organic materials suppress root rot for varying lengths of time. At present, nursery and greenhouse growers successfully use compost-amended potting mixes to suppress soilborne diseases, such as *Pythium* and *Phytophthora* root rots, in container systems (Hoitink et al., 1991). However, limited field studies have been conducted to determine the impact of soil amendments on microbial communities in actual organic and conventional production systems (Drinkwater et al., 1995; Gunapala and Scrow, 1998; Bulluck and Ristaino, 2001). In the case of avocado orchards, organic matter-mediated disease suppression against *Phytophthora cinnamomi* has been observed in avocado agricultural fields organically managed in Australia. Organic amendments (barley straw, sorghum residues, and native grass) were added to the soil under the trees as a mulch layer resulting in the suppression of *Phytophthora* root rot of avocado (Malajczuk, 1979, 1983). Additionally, our previous studies also demonstrated that different organic amendments can influence the composition and diversity of soil bacterial communities in avocado plants growing in microcosms after DGGE analysis, showing enhancement of specific populations such as *Burkholderia* and *Frateuria* (Bonilla et al., 2012a, 2015). Among different organic matter tested on avocado crops, composted almond shells (AS; commercial almond shells derived from the almond industry were piled and traditionally composted) exhibited enhancement of soil suppressiveness against *R. necatrix* (Bonilla et al., 2012a, 2015), the causal agent of avocado white root rot (Pliego et al., 2012). Even when soil suppressiveness against *R. necatrix* is improved after the addition of AS, only subtle changes in the bacterial community and composition and specific enzymatic activities have been reported using DGGE analysis (Bonilla et al., 2015). It must be considered that a wide range of factors can affect soil microorganism communities (van Veen et al., 1997). The soil samples used in our study came from the same orchard (same type of soil, environmental conditions, plant age, and cultivar, etc.), but were under different management, and this was assumed to be the only difference between the samples. The soil influenced by the amendment of AS showed some characteristics that differed from the conventional unamended soil. The almond shells are a high dry matter-containing substrate, composed of approximately 95% organic matter, with poor values of glucose, fructose, or sucrose. The characteristics and composition of AS makes this substrate an acceptable growing media for soilless culture (Valverde et al., 2013). Moreover, it must be taken into account that the avocado is a shallow rooted tree, with most of the feeder roots allocated in the top 15 cm, which needs good aeration. Roots are helped by the presence of a rich surface of organic mulch, as shown by the tendency of healthy feeder roots to grow into any decomposing litter layer (Chanderbali et al., 2013).

In this work, a metagenomic approach to the community composition of amended and unamended avocado soils have been performed for the first time. The use of metabarcoding and GeoChip techniques allowed a better knowledge on the community composition and their potential activities. In first place, an attempt to identify key factors involved in this enhanced suppressivity after the addition of organic amendments revealed the crucial role of the microbiota present in the organic amended soil. The microbiota evolved in the composted almond shells and was crucial for suppressiveness because the reduction of the bacterial population after a heat treatment in the organic amendment resulted in a more conducive phenotype (heat-treated soil samples harbor 10^5^ cfu/g, most likely composed mainly by sporulated bacterial and fungal microorganisms). Moreover, total or partial suppressiveness was recovered when these heat-treated soil samples were complemented with a portion of soil influenced by AS, but it remained conducive when complemented with a portion of conventional soil (CT). This effect has been previously described for different suppressive soils, where sterilization by autoclaving, steam pasteurization, and irradiation rendered soils conducive to the pathogen studied (Malajczuk, 1983; Weller et al., 2002; Mendes et al., 2011). Suppressiveness experiments performed do not excluded the possibility that the disinfected avocado root used could harbor endophytic microorganisms, but our results significantly pointed out the role of the composted almond shells in the plant protection against *R. necatrix*. Thus, our results support the crucial role of microbes present in AS for turning the conducive CT soil into a more suppressive soil against *R. necatrix*.

To gain insights into the microbial diversity present in the soil samples, we used several different approaches. Phylogenetic marker analysis based on the sequencing of 16S rDNA and ITSs revealed a relatively similar array of prokaryotic and eukaryotic populations present in the AS and CT soil samples; however, a different response has been described in the literature for other types of organic matter from different sources, such as composted municipal waste (Zaccardelli et al., 2013). It is remarkable that in our model system, the group of fast-growing, easily cultivable *Proteobacteria* is the dominant group of prokaryotes in both soil samples. These data are similar to those previously observed for other soil and rhizosphere samples with a high presence of organic matter (Lynch and Whipps, 1990; Paul and Clark, 1996; Hawkes et al., 2007; Mendes et al., 2011). Moreover, the representation of the other phyla different than *Proteobacteria* were quite similar among AS-amended and unamended soils, thus contradicting the idea that a highly specific community is stimulated by the addition of AS. Diversity analysis confirmed the previously obtained results (Bonilla et al., 2015), highlighting the enhancement of specific microbial populations in AS-amended samples, such as *Betaproteobacteria* (*Burkholderiales*) and the class of *Gammaproteobacteria*, which have been reported to protect plants from fungal infections in other suppressive soils (Mendes et al., 2011). It is important to note the clear enhancement in AS-amended soil of the order *Steroidobacter*, previously reported to play an essential role in the positive interactions with plants; for example, controlling seed germination, stem, and root elongation or stress protection in plants (Zarraonaindia et al., 2015).

In contrast, analysis of eukaryotic ITS revealed a different abundance distribution of microbes among the two types of soil samples. Fungal clones were the most common and dominant microbial eukaryotes in the soil. AS-amended soil samples had an increased relative abundance of *Ascomycota*. This fact is not surprising considering that *Ascomycetes* are the largest group on true fungi (Larena et al., 1999). Moreover, the dominance of *Ascomycota* has been observed during different composting processes (De Gannes et al., 2013; Neher et al., 2013), where most of them are saprophytic and live on dead organic material that they help decompose (Agrios, 1997; Viebahn et al., 2005). This behavior easily explains their higher abundance when composted almond shells are added to the soil as mulch. Within *Ascomycota*, the group that exhibited the most apparent and highest increase of abundance in AS-amended soil samples was the fungal class of *Dothideomycetes*. A high abundance of *Dothideomycetes* in soils with at high hydrocarbon concentrations has been previously reported (Ferrari et al., 2011), suggesting its preference for those habitats with a high concentration of organic matter where it participates in biomass conversion (Shrestha et al., 2011). Moreover, the large increase of the phylum *Pleosporales* (*Dothideomycetes*) is also not surprising because this group is very well-known to contain species that chlorinate lignin as a first step of biomass conversion during plant litter degradation (Ortíz-Bermúdez et al., 2007). Interestingly, it has been shown that several genera of *Dothideomycetes* exhibit an increased presence in suppressive soils because they harbor endohyphal bacteria from groups that are capable of hydrocarbon biodegradation, such as the *Xanthomonadales, Pseudomonadales, Burkholderiales*, and *Sphingomonadales* (Hoffman and Arnold, 2010). *Dothideomycetes* have also been shown to increase slightly in AS-amended soils. However, the group that shows an apparent decrease in AS-amended soils is *Mortierellales*. This group has a complex phylogeny (Wagner et al., 2013) and is considered to be ubiquitous in the bulk and rhizospheric soil, implying that it could play a role in maintenance of the micro-ecological balance (Miao et al., in press). Interestingly, the group of *Glomeromycota*, which contains different groups of symbiotic fungi previously detected in avocado (Hass and Menge, 1990; González-Cortés et al., 2012), it is clearly detected in unamended soils, but decreased in the amended ones (below 1%). A possible explanation could be that in the AS amended soils, take place a strong competition with other decomposing fungi, such as the *Dothideomycetes*, more adapted to an environment with high amount of decomposing organic matter. Finally, it should be noted that members of *Xylariaceae*, to which *R. necatrix* belongs (Pliego et al., 2012), are less abundant in AS-amended soils, thus revealing a negative effect on this fungal group. These results indicate that the soil fungal community was affected by the soil amendment with AS.

Phylogenetic markers such as the prokaryotic 16S and eukaryotic ITS region do not carry explicit functional information. For this, the use of GeoChip-based analysis allowed for the analysis of microbial functional genes encoding key enzymes involved in major biogeochemical processes that facilitate linking microbial community structure to potential ecological functions (Torsvik and Ovreas, 2002). Using this technique, we screened potential functional gene diversity among unamended and AS-amended soil samples.

Probe signals and DCA analysis indicated that the microbial community functional structures differed between CT and AS soil samples. The sample sites are very close together, so the differences observed in the microbial communities are thought to be the result of amendment with organic matter.

Generally, similar abundance patterns of functional genes involved in nutrient cycling processes such a nitrogen, phosphorous or sulfur cycling, were found in both types of samples. However, AS-amended samples had higher signal intensities for C degradation (carbon cycle) genes than CT, with some differences being statistically significant. Substrates for this group of genes ranged from labile C to more recalcitrant C (e.g., starch, hemicelluloses, cellulose, chitin, and lignin). These results suggest that AS-amended microbial have a greater capacity for C degradation than CT communities. This suggests, as expected, an important role of carbon cycling in response to the addition of organic matter to the soil. However, no differences in gene abundance for N, P, or S cycling was observed. This can be explained because almond shells are a lignin-rich waste resulting from the almond industry, mostly composed of approximately 27% lignin and 73% holocellulose (Caballero et al., 1996), and those cycles were not compromised. However, statistical differences in the abundance of genes related to organic remediation and metal resistance were observed in AS-amended soil displaying lower levels than CT. This observation may be due to a decrease in the available compounds due to the high sorption ability of the composted almond shells and derivate compounds from its degradation, which have been previously reported to be able to remove such substances from the soil (Pehlivan et al., 2009).

Interestingly, both soil samples shared a core of probes corresponding to approximately 90% of the assayed sequences (27364 probes). However, approximately 10% of the total probes analyzed were unique for AS-amended samples (2766 probes). When the sequence of these probes were analyzed, they resulted in a very similar distribution to that previously shown for the whole GeoChip analysis, with above 34.5% corresponding to C cycling, followed by probes related to organic remediation (14.5%), stress (13.4%), metal resistance (11.9%), or the N cycle (8.6%). These results support the following previously described results: systems associated with organic matter-mediated general suppression; suppression typically occurs as a result of the activation of the indigenous microbial community (Lockwood, 1990); and suppressive activities can be generated by one to few populations of organisms (Gerlagh, 1968; Cook and Baker, 1983; Hoitink and Boehm, 1999; Weller et al., 2002). Postma et al. (2000) found that qualitative rather than quantitative shifts in the bacterial community correlate with disease suppressiveness, and several studies indicated that mechanisms within the microbial activity of the soil are responsible for the suppression of pathogens (Rovira and Wildermuth, 1981; Nitta, 1991; Workneh and van Bruggen, 1994; van Os and van Ginkel, 2001).

Among the specific taxa stimulated, *Pseudomonadaceae, Burkholderiaceae, Xanthomonadales*, and *Actinobacteria*, harbor genera and species with activity against plant pathogenic fungi (Postma et al., 2010). Additionally, it is important to note that *Pseudomonas, Rhizobium, Bacillus, Variovorax, Phyllobacterium*, and *Azospirillum*, are considered the most efficient plant growth-promoting bacteria (Bertrand et al., 2001).

Sequencing of specific probes present in AS-amended soils revealed the presence in such soil samples of genes for bacterial and fungal catalases, phenazine biosynthetic genes (from *Proteobacteria*) or the presence of potential antibiotics produced by *Actinobacteria* (data not shown). Nearly all of these probes corresponded to the GeoChip category “soil benefit,” where the antimicrobials from different groups were analyzed. To the best of our knowledge, no probes from *Bacilli* were used, so the role of antimicrobials such as iturin or fengicins, produced by *Bacillus* spp., cannot be discussed based on our results.

It is important to note that the genus *Pseudomonas* (class *Gammaproteobacteria*) and *Bacillus* (class *Bacilli*) are two of the most prominent bacteria that can be isolated from avocado soil and rhizosphere displaying antifungal activity and plant protection against soil-borne pathogens (Cazorla et al., 2006, 2007; González-Sánchez et al., 2010). Our results reinforce the importance of such microorganisms in the soil and root ecology of the avocado crop. These groups of microorganisms can produce metabolites, such as siderophores and antibiotics, with specific suppressive activity against soilborne pathogens. Antagonistic pseudomonads, including *Pseudomonas chlororaphis*, play a role in white root rot suppressiveness (Cazorla et al., 2006; Calderón et al., 2014). However, other types of rhizobacterial taxa may differ in prevalence between suppressive and conducive soils, suggesting that the microbial basis of white root rot could be far more complex than solely a *Pseudomonas* property; it has also been observed for other pathosystems such as *Thielaviospsis basicola*-mediated black root rot of tobacco (Almario et al., 2014).

In conclusion, and taking together the results obtained in this work and in previous works related, a theoretical model about the role of the microorganisms in enhancing suppressiveness after amendment with composted almond shells can be proposed (Figure S5). Soil amendments with composted almond shells resulted in an extra input of organic matter rich in lignin that could be initially degraded by fungal members of the community (such as *Dothideomycetes*) and Actinobacterias. Lignin degradation from composting almond shells would produce a progressive release to the soil of more simple compounds. Those compounds, together with others also present in the almond shells, could lead to an increase in carbon sources available, such as cellulose, hemicellulose, and aromatic compounds. At this point, some *Proteobacteria* already present in the soil (such as *Gammaproteobacteria* and *Betaproteobacteria*) could take advantage metabolizing that available organic matter, thus slightly enhancing their population. These groups of microorganisms could harbor, among other, genes involved in antifungal enzymatic activities and production of antimicrobial compounds that could have an effect on the interaction with other microbes. The resulting modified microbiota after addition of composted almond shells could be more active against some groups of phytopathogenic fungi (as *Xilariales*, where *R. necatrix* is included) finally showing a phenotype of induced suppressiveness effect.

## Author contributions

FC and AD designed and corrected the manuscript. NB contributed to data management. CV performed date management and wrote the manuscript.

### Conflict of interest statement

The authors declare that the research was conducted in the absence of any commercial or financial relationships that could be construed as a potential conflict of interest.
