# Supplementary material for: Microbial Profiling of a Suppressiveness-Induced Agricultural Soil Amended with Composted Almond Shells
Source: Front Microbiol. 2016 Jan 22;7:4. doi: 10.3389/fmicb.2016.00004 (PMC4722121; doi:10.3389/fmicb.2016.00004)
Supplement: Supplementary file 1 [file Table1.pdf]

Table S1.: Assigned function and identity (lineage at class level) of the unique genes detected in AS sample. Classes (bacterial and fungal) related with functional gene categories of unique detected genes of amended soil (AS). R.A. relative abundance.

**Gene Category**	**Class**		**R.A (%)**
**Carbon cycling**	**Bacteria**	**Fungi**	**34.49**
Carbon fixation	*Actinobacteria; Bacteroidia; Betaproteobacteria*		
Starch	*Thermoprotei; Thermoccoci; Thermoplasmata; Actinobacteria; Bacteroidia; Chlamydiia; Chrococcaceae; Bacilli; Clostridia; Deltaproteobacteria; Gammaproteobacteria; Synergistia; Thermotogae*		
Hemicellulose	*Bacilli; Clostridia; Gammaproteobacteria*		
Cellulose	*Bacilli; Clostridia; Betaproteobacteria*	*Neocallimastigomycetes*	
Chitin	*Bacilli; Gammaproteobacteria*		
Lignin		*Agaricomycetes; Dacrymycetes*	
Others Carbon cycling	*Bacilli; Negativicutes; Fusobacteriia; Betaproteobacteria; Epsilonproteobacteria; Gammaproteobacteria*	*Orbiliomycetes; Saccharomycetes; Sordariomycetes*	
**Organic remediation**			**14.53**
Aromatic carboxiyic acids	*Actinobacteria; Dehalococcoidetes; Bacilli*		
Herbicides related compounds	*Clostridia; Alphaproteobacteria; Betaproteobacteria; Deltaproteobacteria*		
Others aromatics	*Gammaproteobacteria*	*Saccharomycetes*	
**Stress**			**13.38**
Oxygen stress	*Gammaproteobacteria*		
Phosphate limitation stress	*Bacilli; Clostridia; Alphaproteobacteria;*		
Heat shock	*Sphingobacteriia*		
Osmotic stress	*Deferribacteres*		

Gene Category	Class		R.A (%)
**Metal Resistance**	**Bacteria**	**Fungi**	**11.86**
Copper<br>Tellurium<br>Arsenic<br>Chromium<br>Others Metal resistance	*Bacilli*<br>*Alphaproteobacteria; Gammaproteobacteria;*<br>*Betaproteobacteria;*<br>*Gammaproteobacteria; Mollicutes*<br>*Methanobacteria; Actinobacteria;*<br>*Dehalococcoidetes; Clostridia;*		
**Nitrogen**			**8.57**
Ammonification<br>Denitrification<br>Nitrogen fixation<br>Nitrogen cycle others	*Bacilli*<br>*Bacilli; Alphaproteobacteria;*<br>*Clostridia*<br>*Epsilonproteobacteria; Deltaproteobacteria;*<br>*Spirochaetia;*		
**Sulphur**			**5.60**
sulfite reductase	*Acidobacteria; Alphaproteobacteria;*<br>*Epsilonproteobacteria; Deltaproteobacteria;*<br>*Gammaproteobacteria*	*Coscinodiscophyceae*	
**Fungi function**			**3.69**
Metal resistance<br>Fungi Carbon degradation<br>Lignin		*Eurotiomycetes*<br>*Oribiliomycetes; Saccharomycetes;*<br>*Sordariomycetes;*<br>*Agaricomycetes; Dacrymycetes*	
**Soil benefit**			**2.64**
Antimicrobial	*Actinobacteria; Bacilli; Clostridia;*<br>*Gammaproteobacteria*	*Sordariomycetes*	
**Phosphorus**			**2.28**
Phosphorus utilization	*Nostocaceae; Epsilonproteobacteria;*<br>*Gammaproteobacteria;*		
**Virulence**	*Gammaproteobacteria*		**1.88**
**Soil borne pathogen**	*Gammaproteobacteria; Tombusviridae;*<br>*Virgaviridae; Secoviridae*	*Sordariomycestes*	**1.08**
