# Supplementary material for: Microbial Profiling of a Suppressiveness-Induced Agricultural Soil Amended with Composted Almond Shells
Source: Front Microbiol. 2016 Jan 22;7:4. doi: 10.3389/fmicb.2016.00004 (PMC4722121; doi:10.3389/fmicb.2016.00004)
Supplement: Supplementary file 2 [file Image1.pdf]

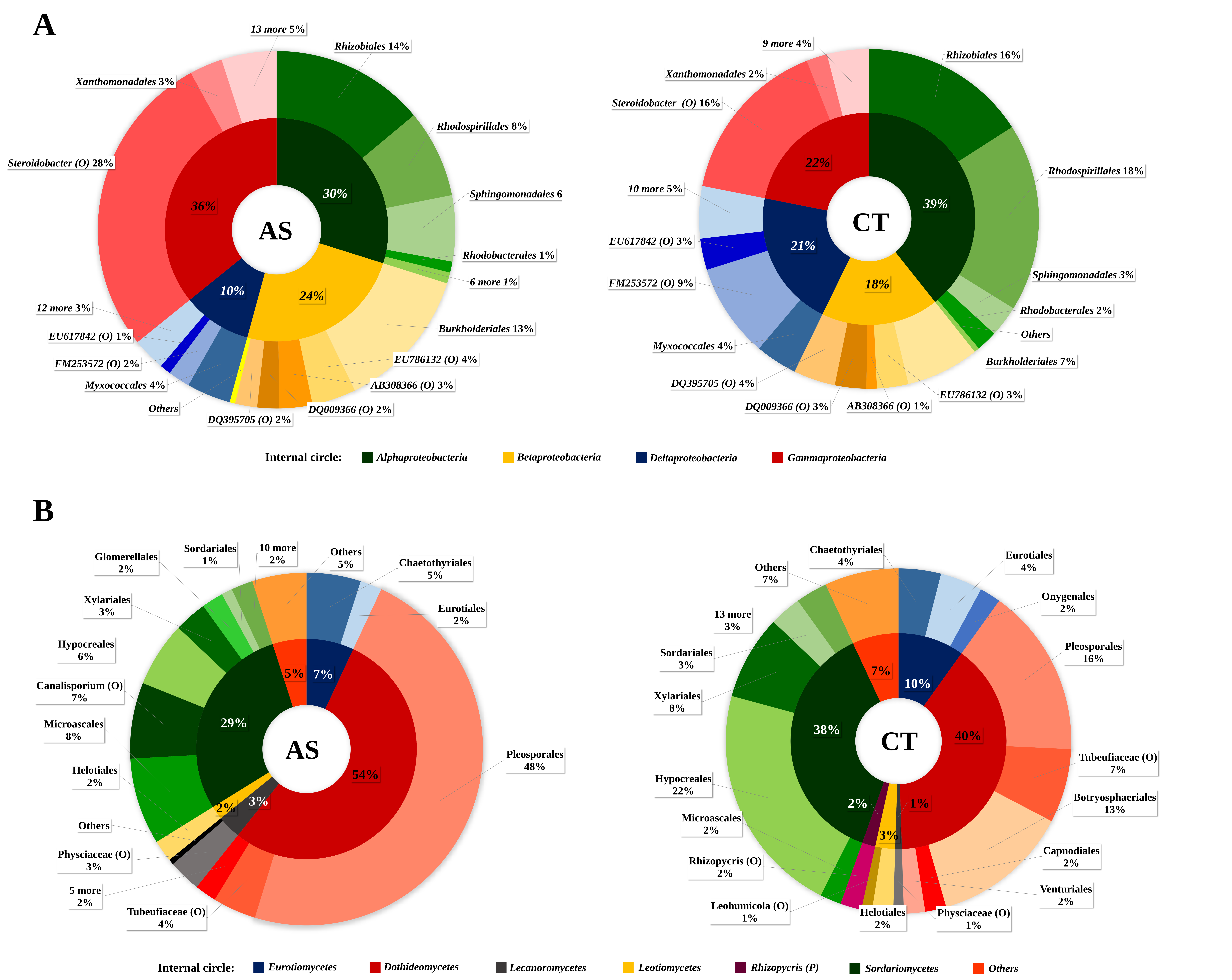

Figure S1.: Microbial community analysis of most represented phyla in each samples. **(A)** Relative abundance (percentage) of different *Proteobacteria* classes (internal circle) and orders (external circle) detected by 16S rRNA gene sequence analysis of soil DNA isolate from amended soil (AS) or conventional soil (CT); **(B)** Relative abundance (percentage) of different *Ascomycota* classes (internal circle) and orders (external circle) detected by ITS region sequence analysis of soil DNA isolate from amended soil (AS) or conventionally managed soil (CT).
