# Supplementary material for: Microbial Profiling of a Suppressiveness-Induced Agricultural Soil Amended with Composted Almond Shells
Source: Front Microbiol. 2016 Jan 22;7:4. doi: 10.3389/fmicb.2016.00004 (PMC4722121; doi:10.3389/fmicb.2016.00004)
Supplement: Supplementary file 3 [file Image2.pdf]

A

16S rRNA	Valid reads	OTUs	Chao1	Coverage (%)	Shannon (H‘)	Simpson (D)
**AS**	3888	1396	2653.34	79.84	6.63	0.0026
**CT**	5046	1578	3037.59	82.46	6.57	0.0035

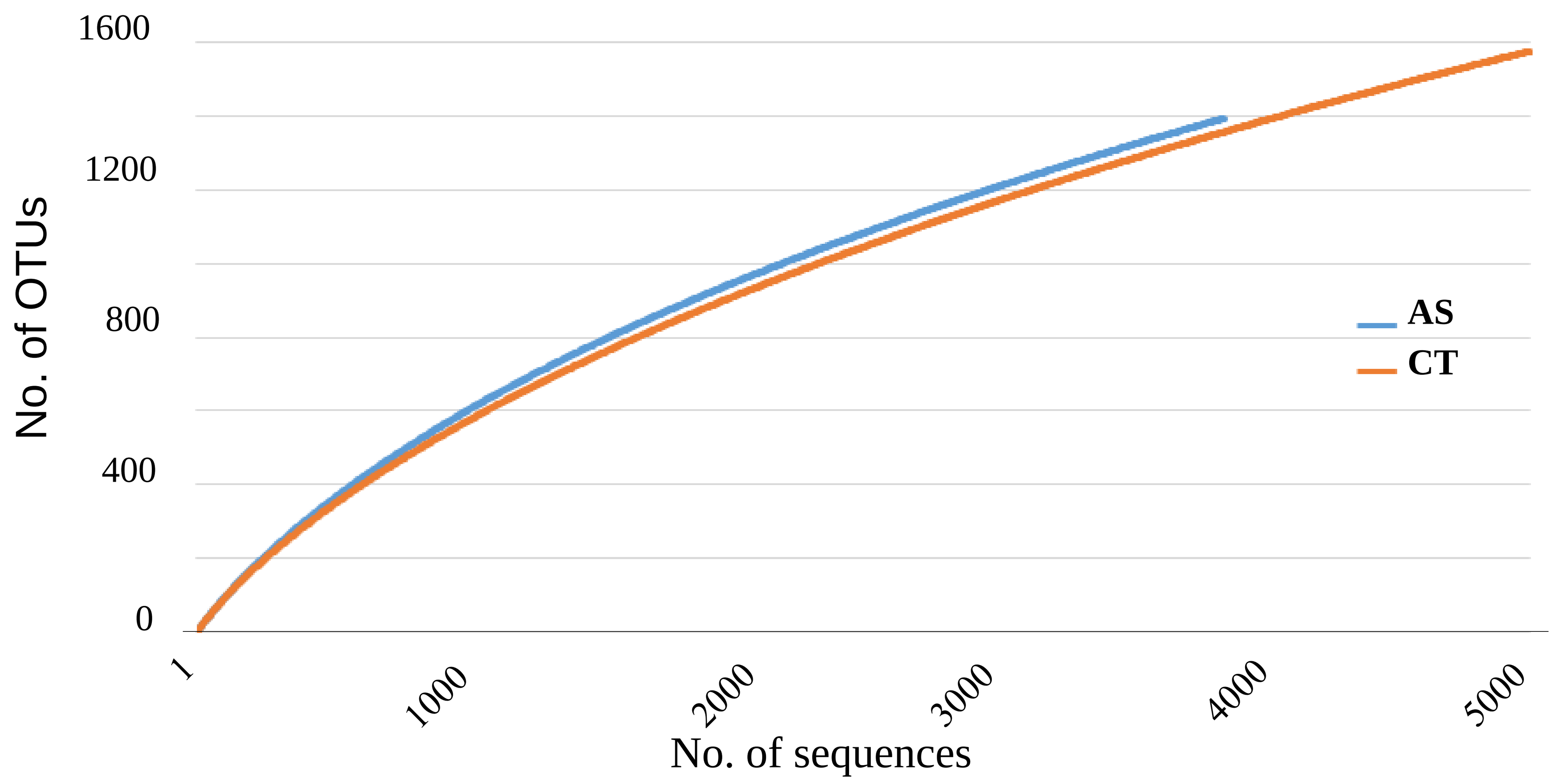

B

ITS	Valid reads	OTUs	Chao1	Coverage (%)	Shannon (H‘)	Simpson (D)
**AS**	6142	664	940.13	96.12	5.27	0.012
**CT**	7288	787	998.53	96.67	5.33	0.017

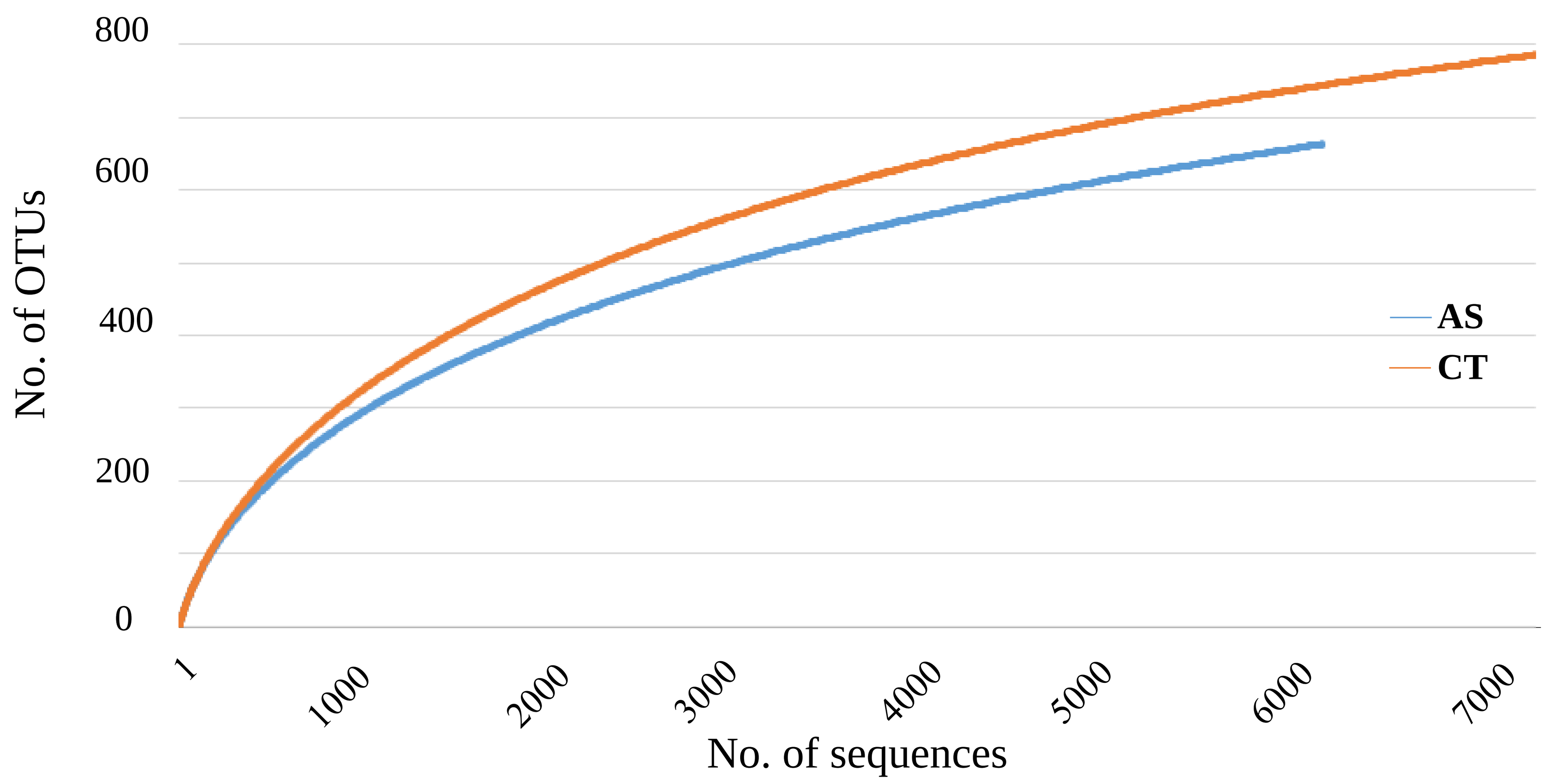

Figure S2.: Quality indixes, alpha-diversity and rarefaction curve of sequencing analysis. (A) Quality indixes (valid reads, OTUs, Chao1 and coverage %) richness (Shannon) , eveness (Simpson) and rarefaction curve obtained of 16S rRNA gene sequence analysis of soil DNA isolate from amended soil (AS) or conventional soil (CT); **(B)** Quality indixes (valid reads, OTUs, Chao1 and coverage%) richness (Shannon) , eveness (Simpson) and rarefaction curve obtained of ITS region sequence analysis of soil DNA isolate from amended soil (AS) or conventionally managed soil (CT).
