# Supplementary material for: Microbial Profiling of a Suppressiveness-Induced Agricultural Soil Amended with Composted Almond Shells
Source: Front Microbiol. 2016 Jan 22;7:4. doi: 10.3389/fmicb.2016.00004 (PMC4722121; doi:10.3389/fmicb.2016.00004)
Supplement: Supplementary file 4 [file Image3.pdf]

A

	AS1	AS2	AS3	CT1	CT2	CT3
**AS1**	-	24364 (77.41)	23686 (75.25)	22665 (66.67)	24157 (65.79)	21790 (61.69)
**AS2**			24829 (78.88)	23074 (66.44)	25405 (69.39)	22920 (64.86)
**AS3**				22908 (66.95)	24545 (66.71)	21671 (60.35)
**CT1**					27185 (76.25)	23424 (65.70)
**CT2**						27639 (77.52)
**CT3**						-
**Num. of detected probes (*)**	27348	28491	27813	29311	33526	29765

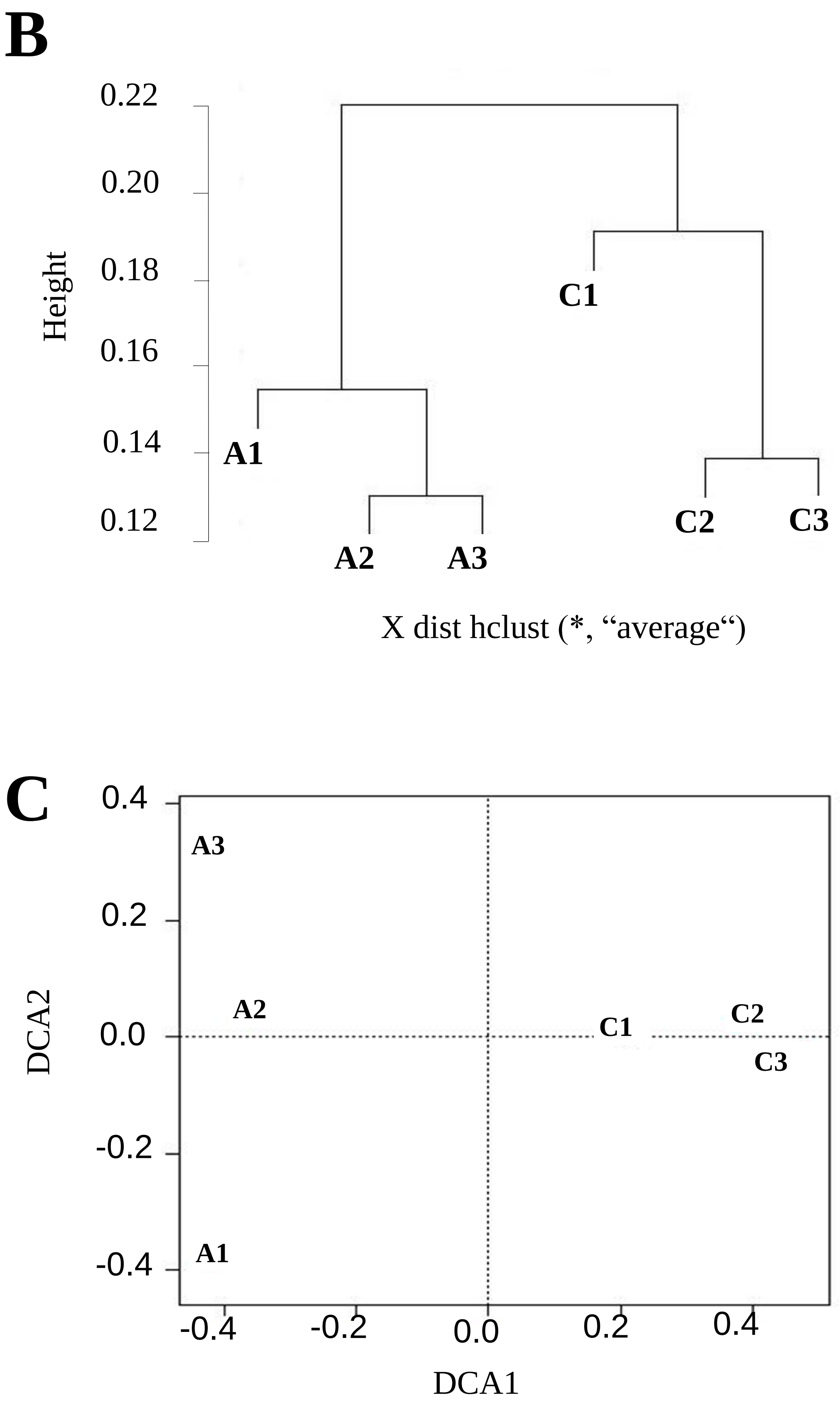

Figure S3.: Unique and overlapped genes, diversity indexes and beta-diversity from GeoChip analysis. **(A)** Summarized data of GeoChip analysis**:** italicized values indicates the number of overlapping genes between samples; values in parentheses shown percentages of overlapping genes between samples; (*) significantly different. **(B)** Bray-Curtis average distance cluster dendogram. **(C)** Detrended correspondance analysis (DCA) of samples from amended soil and convetionally managed soil treatments.
