# Supplementary material for: Microbial Profiling of a Suppressiveness-Induced Agricultural Soil Amended with Composted Almond Shells
Source: Front Microbiol. 2016 Jan 22;7:4. doi: 10.3389/fmicb.2016.00004 (PMC4722121; doi:10.3389/fmicb.2016.00004)
Supplement: Supplementary file 5 [file Image4.pdf]

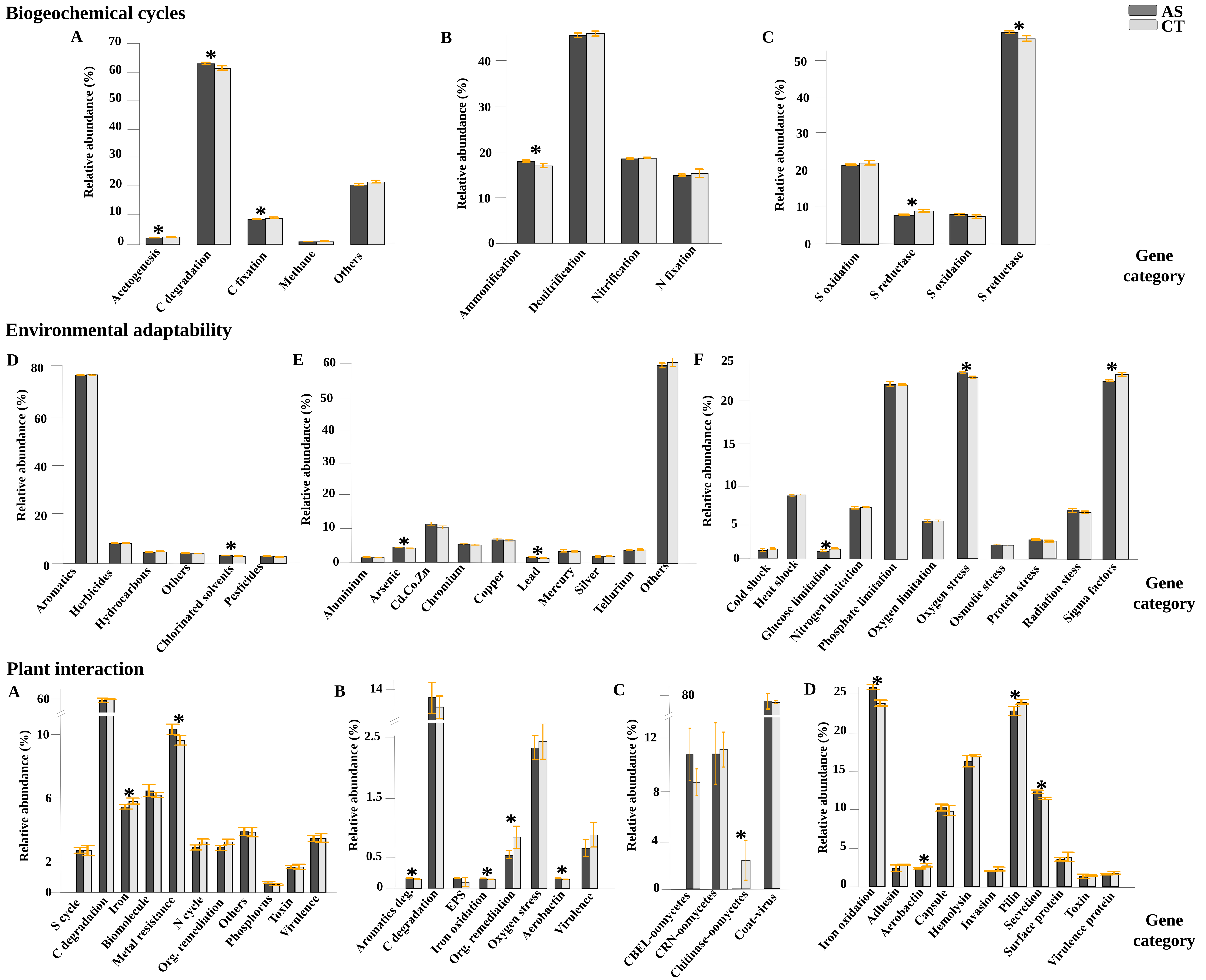

Figure S4.: Biogeochemical cycles analysis. (A) Relative abundance of detected genes from Carbon cycle gene categories **(B)** Relative abundance of detected genes from Nitrogen cycle gene categories **(C)** Relative abundance of detected genes from Sulphur cycle gene categories; **Enviromental adaptability analysis. (D)** Relative abundance of detected genes from different gene categories related with organic compounds degradation **(E)** Relative abundance of detected genes from different gene categories related with metal resistance. **(F)** Relative abundance of detected genes from different gene categories related with stress situations; **Plant interaction analysis. (G)** Relative abundance of detected genes from different gene categories related with fungi functions **(H)** Relative abundance of detected genes from different gene categories related with soil benefits compounds. **(I)** Relative abundance of detected genes from different gene categories related with soil borne pathogens. **(J)** Relative abundance of detected genes from different gene categories related with virulence activity. Asterisc show significant stastitical differences ($P<0.1$) among the two types of soil samples, amended soil (AS) and conventionally managed soil (CT). G.c., gene category. Standard error bars are shown.
