# Supplementary material for: Microbial Profiling of a Suppressiveness-Induced Agricultural Soil Amended with Composted Almond Shells
Source: Front Microbiol. 2016 Jan 22;7:4. doi: 10.3389/fmicb.2016.00004 (PMC4722121; doi:10.3389/fmicb.2016.00004)
Supplement: Supplementary file 6 [file Image5.pdf]

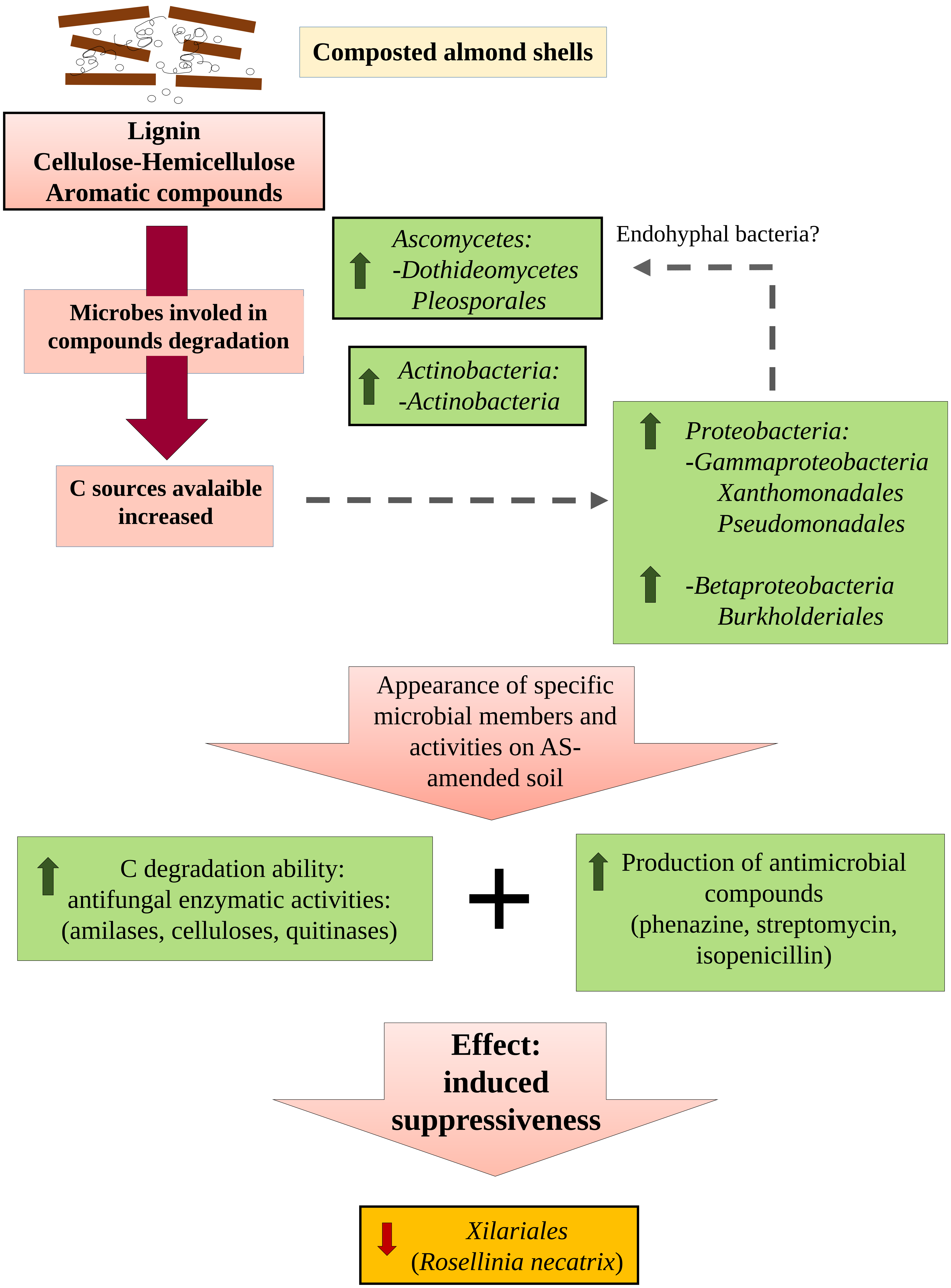

Figure S5.: Hipothetical mode of action of almond shells amendment. Theoretical model proposed about the role of microorganisms in increasing suppressiveness after adition of composted almond shells to agricultural soil. The extra input of organic matter rich in lignin could be degraded by *Ascomycetes* and *Actinobacterias*. An increase in carbon sources available occur and other polysacharides, as cellulose and hemicellulose, and aromatic compounds could be metabolize by *Proteobacteria*. These groups of microorganisms produce enzymes with antifungal activities (such as chitinases) and antimicrobial compounds (such as phenazine). Modified microbiota could have specific activity against fungi pathogens as *Xilariales*, where *Rosellinia necatrix* is included. The effect of microbial community changes induced a suppressiveness in the agricultural soil.
